# Downregulation of Lhx2 Markedly Impairs Wound Healing in Mouse Fetus

**DOI:** 10.3390/biomedicines10092132

**Published:** 2022-08-31

**Authors:** Kento Takaya, Ayano Sunohara, Noriko Aramaki-Hattori, Shigeki Sakai, Keisuke Okabe, Kazuo Kishi

**Affiliations:** Department of Plastic and Reconstructive Surgery, Keio University School of Medicine, Tokyo 160-8582, Japan

**Keywords:** Lhx2, fetal wound healing, skin regeneration, folliculogenesis

## Abstract

Multiple transitions occur in the healing ability of the skin during embryonic development in mice. Embryos up to embryonic day 13 (E13) regenerate completely without a scar after full-thickness wounding. Then, up to E16, dermal structures can be formed, including skin appendages such as hair follicles. However, after E17, wound healing becomes incomplete, and scar formation is triggered. Lhx2 regulates the switch between maintenance and activation of hair follicle stem cells, which are involved in wound healing. Therefore, we investigated the role of Lhx2 in fetal wound healing. Embryos of ICR mice were surgically wounded at E13, E15, and E17, and the expression of Lhx2 along with mitotic (Ki67 and p63) and epidermal differentiation (keratin-10 and loricrin) markers was analyzed. The effect of Lhx2 knockdown on wound healing was observed. Lhx2 expression was not noticed in E13 due to the absence of folliculogenesis but was evident in the epidermal basal layer of E15 and E17 and at the base of E17 wounds, along with Ki67 and p63 expression. Furthermore, Lhx2 knockdown in E15 markedly prolonged wound healing and promoted clear scar formation. Therefore, Lhx2 expression is involved in cell division associated with wound healing and may contribute to scar formation in late embryos.

## 1. Introduction

When adult mammalian skin is wounded, its structure never regenerates and always heals, leaving a scar [1]. No clinical method can completely restore this scar tissue to its original state. However, injured mammalian skin can be completely regenerated at certain embryonic developmental stages [2,3,4]: in the dermis of ICR mice, skin structure is completely restored after injury until embryonic day 16 (E16), but the scar is formed in fetuses and adults after E17. Furthermore, wounds regenerate without a scar, including the texture of the epidermis, up to E13, whereas those of fetuses after E14 leave visible marks [5]. Therapeutic development to achieve complete skin regeneration requires focus on the molecules that change at this transition. Skin development is governed by the interaction between the epithelium and mesenchyme, resulting in the formation of the epidermis and a number of skin appendages, including the hair follicle (HF) [6]. The skin appendages are observed from E14 with condensation of the placode and dermis, HFs appear from E15, and the tissue structure becomes equivalent to that of adult mice from E16 onwards. During postnatal life, the epidermis and HF self-renew and regenerate with the involvement of stem cells that can differentiate into different epithelial cell lineages [7,8]. Epithelial stem cells also contribute to epidermal regeneration after injury [1]. In normal skin, distinct populations of epithelial stem cells contribute differentially to HF and epidermal regeneration during the hair cycle [9]. However, the wound activates a population of epithelial stem cells that normally supply progeny to the HF [10]. Furthermore, a close relationship between HF stem cells and wound healing exists, as re-epithelialization is faster at sites where hairs are present [11].

The Lim-homodomain transcription factor Lhx2 is a key regulator of the switch between stem cell maintenance and activation in HF [12,13]. Lhx2 is expressed in the bulge and papilla of quiescent HF in mice [14], and Lhx2-deficient mice are unable to maintain quiescent bulge stem cells [12]. Lhx2 acts as the center of a gene network that coordinates multiple signals regulating stem cell maintenance, differentiation, and self-renewal as well as cell fate decisions during organ development [15,16,17].

Lhx2 is strongly expressed specifically in the HF of adult mice wounds and is involved in wound healing [12]; however, reports are scanty on its analysis during embryonic development in mice. We have previously reported that fibroblast growth factor and Wnt signaling are involved in wound healing during mouse development [18,19]. Here, we investigated the relevance of Lhx2 expression to the timing of skin regeneration by observing the differential expression of Lhx2 in the wound during embryonic development. Wound healing in mouse fetuses progresses quickly and is fully epithelialized 48–72 h after wounding [5]. Therefore, we focused on wounds 24 h post-injury to observe the molecular mechanisms involved in the wound healing process.

We also examined the effect of external on wound healing. The findings may contribute to the development of therapies that achieve complete skin regeneration.

## 2. Materials and Methods

### 2.1. Ethical Consideration

The research protocol was reviewed and approved by the Institutional Animal Care and Use Committee of the Keio University School of Medicine (approval number: 20170914). All experiments were conducted in accordance with the institutional guidelines for animal experiments at Keio University. This study was reported in accordance with the Reporting of In Vivo Experiments on Animals (ARRIVE) guidelines.

### 2.2. Fetal Wounding Procedure

Eight-week-old female ICR mice were used in this experiment. Mice were obtained from Sankyo Laboratory Services, Inc. (Tokyo, Japan). Vaginal plugs were checked twice daily. When a plug was observed, the fetus was designated E0; on E13, E15, and E17, the fetus was wounded. Surgeries were performed on five pregnant mice per time point. Pregnant mice were anesthetized with 3% isoflurane, and the abdominal wall was incised to expose the uterus. Using an operating microscope, the myometrium and amniotic and yolk sacs were incised. Then, using surgical micro-scissors, a full-layer incision of approximately 2 mm in length was made in the lateral thoracic region of the fetus; on E13, after wounding, the amnion and yolk sac were sutured with 9-0 nylon, but the myometrium was left open and unstitched to prevent uterine rupture owing to internal pressure buildup. The fetus was returned to the abdominal cavity with the amnion and yolk sac covered, but the myometrium was uncovered, and the abdomen was closed; on E15 and E17, after making the fetal wound, the myometrium was sutured with 9-0 nylon, the uterus was returned to the abdominal cavity, and the abdomen was closed. Then, just before the closure of both wounds, 1 μg/gBW ritodrine hydrochloride (FUJIFILM Wako Pure Chemical Co., Ltd., Osaka, Japan), a uterine relaxant, was intraperitoneally administered. The peritoneum and skin were then sutured with 5-0 nylon thread. Maternal mice were euthanized by cervical dislocation, and fetuses were harvested 24 h after wounding. At each time point, wounds were created in at least four fetuses. Skin samples of fetuses were harvested and fixed in 4% paraformaldehyde for 24 h, and the fixed tissues were embedded in paraffin and stained.

### 2.3. Immunohistochemistry

Paraffin-embedded specimens were sliced into 7 µm-thick sections and mounted on glass slides. After drying overnight at room temperature (15–25 °C) to allow the specimens to adhere to the slides, paraffin was dissolved in a slide heater (ThermoBrite; Leica Biosystems, Nussloch, Germany) at 65 °C for 30 min immediately before use. The slides were then deparaffinized by washing twice with xylene (5 min each) at room temperature. Slides were transferred twice to 100% ethanol (3 min each), once to 95%, 70%, and 50% ethanol (3 min each), and rehydrated at room temperature. After antigen activation by heat, the samples were incubated with 2% goat serum in phosphate-buffered saline (PBS) for 30 min at room temperature to block nonspecific binding sites. Subsequently, they were incubated overnight at 4 °C with the following primary antibodies diluted 1:100 in PBS: anti-Lhx2 (ab78363, Abcam, Cambridge, U.K.), anti-cytokeratin-10 Ab-2 (MS611B, Thermo Fisher Scientific, Waltham, MA, USA), anti-Ki-67 (556003, BD Biosciences, Franklin Lakes, NJ, USA), anti-loricrin (ab24722, Abcam), and anti-p63 (559951, BD Biosciences). After washing thrice with PBS, the cells were incubated with a 1:500 dilution of an anti-mouse horseradish peroxidase-labeled rabbit IgG reagent ImmPRESS (Vector Laboratories Inc., Newark, CA, USA) in PBS for 1 h at room temperature. Signals were amplified using the avidin-biotinylated peroxidase complex method using a VECTASTAIN ABC kit (Vector Laboratories) and incubated in a 20 mg/dL 3,3′-diaminobenzidine solution (FUJIFILM Wako Pure Chemical Co.) for 1–3 min. The sections were then washed once for 5 min with running tap water before counterstaining the nuclei with Gill’s hematoxylin solution (Merck Millipore, Billerica, MA, USA) for 6 sec at room temperature. Finally, sections were rinsed with tap water for 5 min, dehydrated with ethanol (95%, 95%, 100%, and 100%, 5 min each) four times, rinsed with xylene thrice, and sealed with Mount Quick Sealant (TakaraBio, Shiga, Japan).

Slides were observed using an integrated stereomicroscope (BZ-X800; KEYENCE, Osaka, Japan).

### 2.4. In Situ Hybridization

In situ hybridization analysis was performed using a QuantiGene ViewRNA ISH Tissue Assay kit (Thermo Fisher Scientific) according to the manufacturer’s protocol using an antibody against Lhx2 (VB1-14743, Thermo Fisher Scientific). Optimization conditions were 5 min for boiling and 20 min for enzymatic treatment.

### 2.5. Laser Microdissection (LMD), RNA Isolation, and Reverse Transcription

LMD was performed using a PALM MicroBeam (Carl Zeiss, Oberkochen, Germany). Manufacturer-recommended slide and collection tubes (AdhesiveCap 500 opaque, Carl Zeiss) were set up, and after adjusting the aperture and intensity using a 20× magnification objective lens, only the epidermis and dermis structures of the wound were carefully cut from the tissue. Tube caps were filled with Buffer RLT (RNeasy Micro Kit, Qiagen, Hilden, Germany) with β-mercaptoethanol to allow isolation of intact RNA. Total RNA was extracted from cells or skin tissues using a monophasic solution of phenol and guanidine isothiocyanate (ISOGEN; NipponGene, Tokyo, Japan) according to the manufacturer’s instructions. Total RNA was mixed with random primers, reverse transcriptase, and dNTP mixture (TakaraBio) and incubated in a T100TM thermal cycler (Bio-Rad Laboratories, Inc., Hercules, CA, USA) at 25 °C for 5 min, 55 °C for 10 min, and 80 °C for 10 min to produce cDNA.

### 2.6. Quantitative Real-Time Polymerase Chain Reaction (RT-qPCR)

RT-qPCR was performed using an Applied Biosystems 7500 Fast Real-Time PCR System (Thermo Fisher Scientific). A total of 40 cycles were performed, and the fluorescence of each sample was measured at the end of each cycle. The PCR was performed in two major steps: holding the reagent at 95 °C for 3 s (denaturation) and at 60 °C for 30 s (annealing and extension). In the subsequent melting curve analysis phase, the temperature was increased from 60 to 95 °C, and fluorescence was continuously measured. Primers against Lhx2 (Assay ID: Mm00839783_m1) were used; PCR master mix (Cat. 4352042; Applied Biosystems, Foster City, CA, USA) was used according to manufacturer’s instructions; *ACTB* (Mm02619580_g1) was used as a control for normalization according to the manufacturer’s instructions. Gene expression levels at normal sites were used as a baseline, and fold-changes were determined using the 2^−ΔΔCt^ method.

### 2.7. siRNA Experiment for Mouse Fetuses

E15 mice were treated with siRNA reagents in the amniotic fluid after normal fetal surgery. The Lhx2 siRNA reagent (156112 and 156113, Thermo Fisher Scientific) was used at 3 mg/mL, approximately 200 µM concentration. Equal amounts of complex buffer (1377501, Thermo Fisher Scientific) and siRNA were mixed with a double volume of invivofectamine (1377501, Thermo Fisher Scientific) to prepare the reagent. Negative siRNA (Silencer™ Negative Control No. 1 siRNA, Thermo Fisher Scientific) was used as a negative control. The prepared reagents were then incubated at 50 °C for 30 min, placed in a Float-A-Lyzer dialyzer, and dialyzed with 1 L of PBS (pH 7.4). The final concentration of siRNA preparation reagent, which was approximately 0.35 mg/mL, was administered in approximately 40 µL of amniotic fluid per fetal mouse. After 72 h, tissues were harvested, and the paraffin sections were stained with hematoxylin and eosin.

### 2.8. Statistical Analysis

Mann–Whitney *U* tests were performed to determine the significance of differences in gene expression using the Statistica software version 9.0 (StatSoft, Tulsa, OK, USA). Results of descriptive statistics are presented as mean ± standard deviation. The threshold for statistical significance was set at *p* < 0.05. Each experiment was performed in triplicate.

## 3. Results

### 3.1. Lhx2 Expression Is Enhanced in the E17 Wound

**Figure 1:**
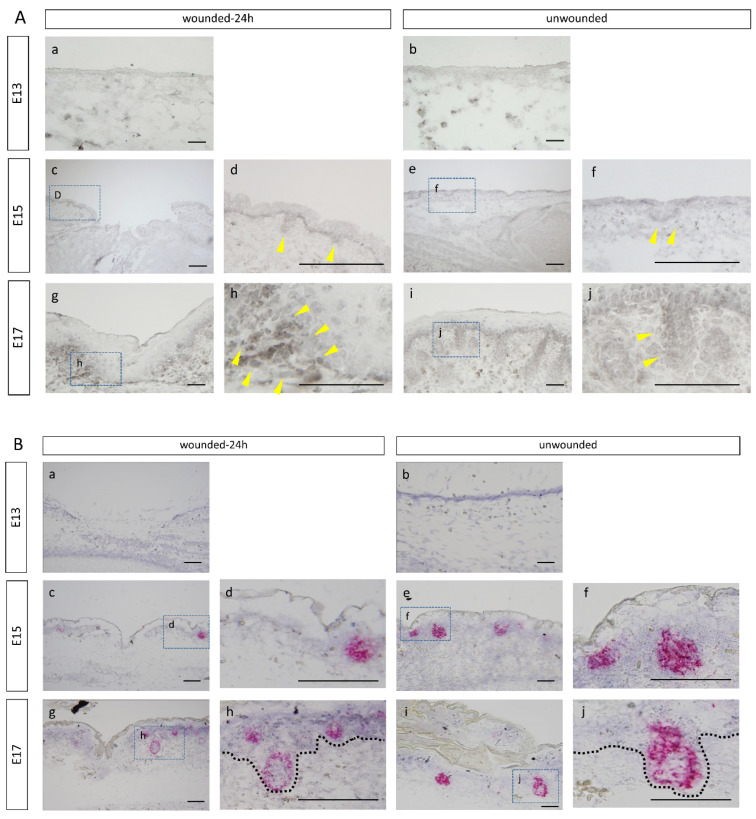

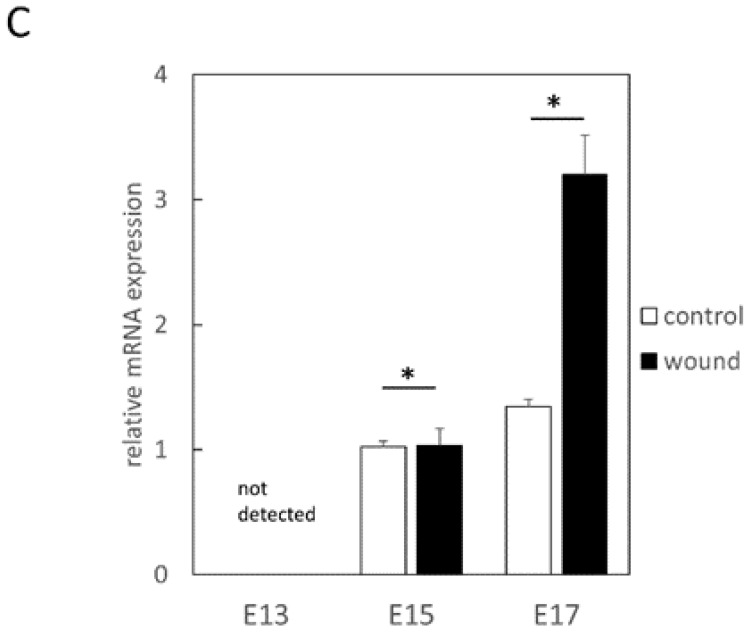
Lhx2 expression in the wound during embryonic mouse development. (**A**) Immunostaining. Yellow arrows: expression sites, Bar = 100 µm. (**a**–**c**,**e**,**g**,**i**): low magnification images of the wound; (**d**,**f**,**h**,**j**): high magnification images of the skin appendages. (**B**) In situ hybridization. Expression is specific to the hair follicles of E15 and E17 and the dermis of the E17 wound. Bar = 100 µm. (**a**–**c**,**e**,**g**,**i**): low magnification images of the wound; (**d**,**f**,**h**,**j**): high magnification images of the skin appendages. (**C**) Gene expression was significantly upregulated in the E17 wound compared to that in normal skin. * *p* < 0.05. Scale bar = 100 μm.

To investigate Lhx2 expression during wound healing in fetal mouse embryos, we observed wounds 24 h after wounding, i.e., when re-epithelialization was not complete. Immunostaining results showed that at E15 and E17, Lhx2 was strongly expressed in follicles and weakly distributed throughout the epidermal basal layer in the wound and normal areas (Figure 1A). At embryonic day 13, expression could not be confirmed owing to the absence of follicle formation during the developmental stage. Importantly, on E17, Lhx2 was found to be expressed not only in HFs but also in the dermis cells of the wound. Similar to the immunostaining results, high expression of Lhx2 mRNA was observed in HFs of normal skin in E15 and E17. In E15, there was no difference in Lhx2 mRNA expression levels between wounded and normal areas; however, in E17, Lhx2 mRNA expression was enhanced in the dermis of the wound area compared to that in normal areas (Figure 1B). Real-time PCR showed no difference in Lhx2 expression in epidermis and dermis between normal skin and the wound on E15; however, the expression increased in the wound at E17 (*p* = 0.0008) (Figure 1C).

### 3.2. Lhx2 Is Associated with Cell Division in the Fetal Wound

**Figure 2:**
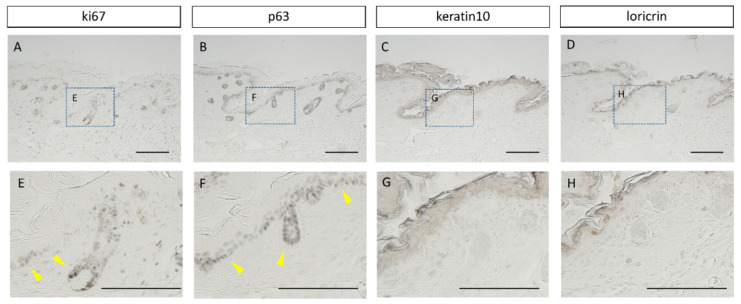
Relationship between cell division/differentiation and Lhx2. (**A**–**D**): low magnification images of the wound; (**E**–**H**): high magnification images of the skin appendages. Lhx2-rich follicles and epidermal basal layer expressed Ki67 and p63 and actively underwent cell division. Scale bar = 100 μm.

To investigate the relationship of Lhx2 with cell differentiation and division in the wound, immunohistochemical analysis of the respective markers Ki67, p63, keratin-10, and loricrin was performed in E17 wounds (Figure 2). The results showed that follicles with high Lhx2 expression also expressed Ki67 and p63, indicating active cell division. The results showed that follicles and the epidermal basal layer with high Lhx2 expression also expressed Ki67 and p63, indicating active cell division. In layers of the epidermis, where differentiation had progressed and cell division had declined away from the basal layer, Ki67 and p63 were not expressed. Therefore, Lhx2 is associated with cell proliferation during fetal wound healing.

### 3.3. Lhx2 Knockdown Inhibits Fetal Wound Healing

**Figure 3:**
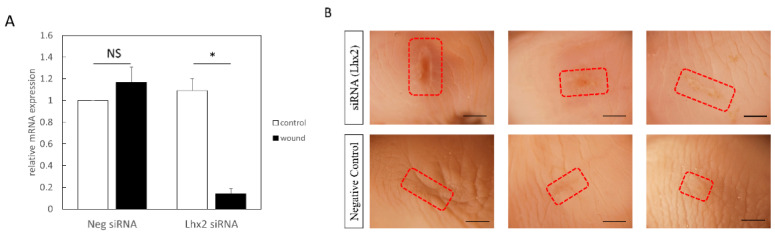

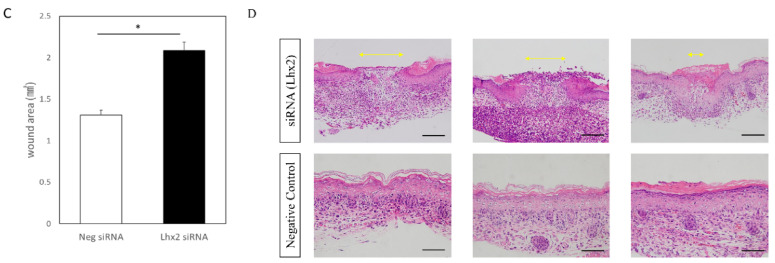
Effect of Lhx2 knockdown on fetal wound healing. (**A**) siRNA-mediated Lhx2 knockdown. Lhx2 expression was significantly suppressed in the Lhx2 siRNA group. (**B**) Macrograph of the scar, Bar = 500 µm. (**C**) Comparison of the area of the scar, which was significantly large in the Lhx2 siRNA group. (**D**) H-E stained image of the wound. In the Lhx2 siRNA group, wounds were inadequately epithelialized (yellow arrows). Additionally, loss of hair follicles is observed. * *p* < 0.05. NS: non-significant. Scale bar = 100 μm.

Since Lhx2 expression was observed in fetal wound healing, particularly in the late embryonic period, we speculated that Lhx2 inhibition may prolong the wound healing process. Therefore, we knocked down Lhx2 by injecting siRNA into the E15 amniotic fluid. Lhx2 expression was significantly suppressed by siRNA treatment in wounds sampled by laser microdissection (*p* = 0.0023) (Figure 3A). Consequently, wound healing was clearly suppressed in the Lhx2 knockdown group compared to that in the E15-negative control (Figure 3B). Wound size was bigger in the Lhx2 siRNA group than in the control (*p* = 0.0074) (Figure 3C). Histologically, Lhx2 knockdown resulted in the loss or reduction in follicles at the wound margins, and the wounds were inadequately epithelialized (Figure 3D).

## 4. Discussion

The repair of skin injury is a complex process, in part owing to the mobilization of distinct populations of undifferentiated progenitor cells from adjacent HFs to the regenerating epidermis [8,20] and reportedly contributes, at least in part, through positive regulation of SRY-Box transcription factor 9 (Sox9) and transcription factor 4 (Tcf4), which play important roles in regulating HF morphogenesis and the maintenance of HF stem cells after birth [21,22]. However, these have been observed in adult animals and have not been reported in fetal mice wherein the wounds are regenerating and HFs are in the developmental stage.

Here, we investigated the relationship between wound repair and Lhx2 using a unique fetal mouse model of wound healing. Similar to previous studies, we observed Lhx2 expression around HF in fetuses. Furthermore, Lhx2 expression was enhanced in cells at the base of the dermis at the E17 wound site. Lhx2 knockdown resulted in a prolonged wound healing, while no Lhx2 expression was observed in E13 owing to insufficient development of the skin appendages, including the HF.

The inflammatory cells or fascia-derived fibroblasts present at the base of the wound in E17 fetuses result in scar formation. We previously observed that dermal mesenchymal cells are responsible for the reconstruction of the dermal structures in the wound, and scar formation in E17 and beyond is mediated by mesenchymal cells of fascial origin [23]. Since then, the classification and behavior of skin mesenchymal cells, i.e., undifferentiated fibroblasts, have been reported to play an important role in wound healing [24,25]. One lineage of fibroblasts forms the upper dermis, including the dermal papilla that regulates hair growth and the erector pili muscle, and other forms the lower dermis, including reticulin fibroblasts, which synthesize most of the fibrous extracellular matrix, and preadipocytes and adipocytes in the subcutaneous tissue [26]. In the case of wounds in adults, the dermal repair is mediated by the lower lineage, and the upper dermal fibroblasts are mobilized only during re-epithelialization. Postnatal β-catenin removal in fibroblasts promoted HF regeneration in neonatal and adult mouse wounds, whereas β-catenin activation reduced HF regeneration in neonatal wounds [27]. Thus, the ability of postnatal epithelialization in wounds reflects increased skin Wnt/β-catenin activation in the wound bed, increasing the number of fibroblasts that cannot induce HF formation [27]; however, loss of Lhx2 is believed to cause misregulation of the Wnt/βcatenin pathway [28], thereby affecting the formation of scar and follicles. However, further investigation on Lhx2 is required because several independent mechanisms regulate the mobilization of distinct HF stem cell populations and mesenchymal cell populations into the regenerating skin at the wound site [29,30].

A limitation of this study is the necessity to examine the effect of Lhx2 knockout on wound healing during developmental stages. Lhx2 knockout (−/−) mice die between E15.5 and E16.5 owing to abnormalities in the development of the liver, brain, and hematopoietic system [31]. Because Lhx2^+/−^ mice are viable, fertile, and have approximately 50% reduced levels of Lhx2 protein and mRNA in skin compared to those of wild-type mice [31], future experiments using this model mouse should be considered. Alternatively, observations of wound healing using conditioned knockout mice such as the PDGFRa-Cre system should be considered. Confirming whether this phenomenon can be applied to mammals other than mice is also necessary. Furthermore, since mast cells and inflammatory cells are intricately involved in wound healing in addition to fibroblasts, the effect of Lhx2 expression on the behavior of these cells must be investigated.

In addition, there is no treatment that completely regenerates scars; however, the decreased expression of Lhx2 in scarless wound healing observed in this study may contribute to the development of scarless treatments.

Based on the present data showing a correlation between Lhx2 and non-scarring skin regeneration during development, its application for human skin may be useful in developing scarless wound healing and regenerative medicine.

## Data Availability

The data presented in this study are available on request from the corresponding author.
